# Does online case-based learning foster clinical reasoning skills? A mixed-methods study

**DOI:** 10.1016/j.fhj.2024.100210

**Published:** 2024-11-13

**Authors:** Jun Jie Lim, Bhavani Veasuvalingam

**Affiliations:** a School of Medicine and Population Health, University of Sheffield, Sheffield, UK; b Faculty of Medical Sciences, Newcastle University Medicine Malaysia, Iskandar Puteri, Johor, Malaysia; c International Medical University Centre for Education, International Medical University, Kuala Lumpur, Malaysia

**Keywords:** Medical education, Clinical education, Clinical reasoning, Case-based discussion, Medical student, Blended learning

## Abstract

**Background:**

Blended learning, integrating face-to-face and virtual methods, has become essential in clinical education, enhancing student satisfaction, engagement and knowledge outcomes. Particularly, online case-based learning emerges as a promising pedagogy to foster clinical reasoning skills. Despite the well-documented clinical reasoning cultivation through face-to-face case-based learning, the ability of online case-based learning to cultivate clinical reasoning remains unexplored. This study investigates the role of online case-based learning in fostering clinical reasoning skills among clinical-year medical students.

**Methods:**

A mixed-methods sequential explanatory research study was adopted. In the first phase, quantitative data were gathered through a 16-item Likert scale questionnaire adapted from validated clinical reasoning questionnaires. In the second phase, focus group discussions were conducted to expand on the understanding of quantitative results.

**Results:**

In total, 160 students completed the questionnaire (45% response rate), and 26 participated in focus group discussions. Participants agreed that online case-based learning fostered clinical reasoning skills (mean = 2.94) through different formats, such as clinical role play, simulated ward rounds and virtual consultation. Compared to face-to-face clinical teaching, the focus group revealed that participants were allowed to practise giving explanations to patients, engage in more in-depth discussions, and receive more comprehensive feedback on their clinical reasoning skills during online case-based learning. The barriers to online clinical reasoning skills development were poorer communication skills development and reduced student engagement. The lack of patient complexities of cases and the inability to perform physical examinations hindered students’ clinical reasoning ability. Suggestions to improve clinical reasoning cultivation include utilising actual patient cases, increasing case complexity and session interactivity.

**Conclusion:**

This study highlights how online case-based learning can support the development of clinical reasoning skills in medical students, encouraging future educators to adopt a blended learning approach. Future research should focus on objective assessments, long-term impacts and innovative methods to improve clinical reasoning skill development continuously.

## Highlights

• Online case-based learning (CBL) supports clinical reasoning (CR) skill development in medical students.
• Benefits include improved question exploration and immediate feedback in CBL sessions.
• Challenges include unrealistic case presentations and decreased student engagement.
• Recommendations suggest incorporating real patient simulations and breakout rooms.
• Further research is needed to assess CR skills and the long-term impact of online CBL.

## Introduction

In this post-pandemic world, blended learning, which integrates face-to-face and virtual learning, has gained acceptance in clinical education as an effective complement to traditional teaching pedagogies.^1^ Systematic reviews reported blended learning as superior to traditional learning in providing better student satisfaction, motivation, engagement, performance, knowledge outcomes and acquisition.^1^, ^2^, ^3^ Furthermore, clinical educators are urged to optimise their climate footprint by balancing educational effectiveness with environmental costs^4^ through optimising the use of virtual learning.^5^^,^^6^ Among various blended learning pedagogies, online case-based learning (CBL), defined as an educational approach that integrates theory and practices through authentic cases to foster diagnostic skills and promote patient-centred learning,^7^^,^^8^ emerged as a prominent educational approach. Underpinned by online collaborative learning theory, online CBL curricula design aims to foster clinical reasoning (CR) by encouraging engagement in virtual case discussions and bridging theoretical knowledge and clinical practice.^9^ There is a wide range of online CBL studies, including interactive online systems,^10^, ^11^, ^12^ virtual clerkships,^13^^,^^14^ virtual patient cases,^15^, ^16^, ^17^, ^18^, ^19^, ^20^ virtual patient simulators^21^, ^22^, ^23^ and virtual patient platforms.^24^ Systematic reviews have found that virtual patients improve data gathering, uncertainty management and overall CR skills.^25^^,^^26^ Furthermore, virtual simulators increase self-efficacy and allow students to achieve higher levels of mastery through deliberate practice;^23^ the same has been proven for virtual bedside teaching that exposes learners to various clinical problems and patient presentations.^27^

CR is defined as a higher-order thinking process that involves gathering, interpreting, integrating and critically evaluating patient data to develop diagnoses and formulate treatment plans.^28^ CR is one of the core elements of workplace knowledge, termed ‘tacit knowledge’,^29^ and has been the main focus of attainment by competency frameworks across healthcare.^30^, ^31^, ^32^, ^33^ CR skills involve assessing a patient’s various symptoms and correctly identifying a diagnosis, with the ultimate objective of formulating a treatment plan to ensure optimum patient care and minimise medical errors.^34^ Understanding CR is important to ensure that the teaching and assessment of CR are efficiently implemented.^33^^,^^35^ Despite the well-documented facilitators^36^, ^37^, ^38^, ^39^, ^40^ and barriers of CR cultivation through face-to-face CBL, the ability of online CBL to foster CR skills among medical students remains unexplored.^8^ Although Duffy *et al*^41^ reported that online CBL developed students’ clinical skills, their focus has been on students’ performance and satisfaction, thereby lacking a focused evaluation of students’ CR skills. Furthermore, although CR assessment instruments such as a CR task checklist^42^ and a Likert-scale questionnaire^43^ have previously been developed, no studies documented their use in assessing students’ CR ability, specifically during online CBL. Early adoption of CBL is a cornerstone in fostering CR, as it encapsulates constructivist, experiential and self-regulated learning methods intended to stimulate and develop higher-order cognitive skills.^9^^,^^44^ Therefore, an in-depth understanding of the ability of online CBL to cultivate CR skills is crucial to guide educators in designing effective online instructional materials and harnessing their benefits in fostering CR skills.^45^

In this study, we explored the ability of online CBL to foster CR skills among medical students by posing the following research question: How effective is online CBL in enhancing CR skills among clinical-year medical students, and what are the perceived barriers and facilitators compared to traditional face-to-face methods?

## Methods

### Contextual background

This study was conducted at Newcastle University Medicine Malaysia, a branch campus of Newcastle University in Malaysia. Our study participants are clinical-year medical students (ie years 3, 4 and 5) who rotate through clinical placements across various primary and secondary healthcare settings. From March 2020 to June 2021, clinical rotations were interrupted, and all teachings were transitioned to online tutor-led CBL with intermittent and phased return to face-to-face for 16 months. The online CBL is a tutor-led small group teaching on a patient case chosen by the tutor, which takes place entirely on Zoom.

### Data collection

We adopted a mixed-method sequential explanatory study design first to quantify students’ CR ability and then refine and deepen our understanding by exploring participants’ views through subsequent qualitative analysis. In the first phase, we purposively sampled clinical-year medical students (n = 354) as they allow for detailed and contextually rich exploration due to their extensive experience with online CBL through email. Students who provided written consent were sent a validated web-based online questionnaire and focus group invitation. The quantitative results were then analysed to identify areas needing further elaboration. In the second phase, we conducted online focus group discussions to explore students’ perspectives in greater depth in those areas identified by the questionnaire. By using mixed methods, we could gain a better understanding of the role of online CBL in fostering CR skills through the triangulation and corroboration of the quantitative and qualitative results for interrelationships.^46^

### Questionnaire

We adapted our questionnaire from previously validated CR assessment instruments in the literature, (i) Clinical Reasoning Indicators-History Taking-Scale (CRI-HT-S) and (ii) Goldszmidt’s CR task checklist^42^ (See Appendix A - Questionnaire). The questionnaire consists of 16 items, divided into three sections: (A) history taking, (B) case discussion (C) investigations and management. Cronbach’s alpha coefficients for every item were above 0.8, which was considered satisfactory. All corrected item total corrections value were higher than 0.35, which allowed all items to be included in the instrument. Answers to statements are graded using a 4-point Likert scale (1 = strongly disagree to 4 = strongly agree). To discern the difference between perceptions of CR ability between early (year 3) and senior (year 5) clinical year students, the statistical comparison was made using the Spearman correlation. A *p* value <0.05 was considered significant.

### Focus groups

In the second phase, we have chosen to conduct semi-structured focus group discussions (FGDs) to not only explore the elements of online CBL that foster CR, but also utilise the interactions between participants to generate data and explore metacognitive thought processes^47^ (See Appendix B – Semi-structured interview guide). To mitigate the effect of power imbalances between students of different year groups, BV divided students into separate focus groups according to their year group.^48^ Participants are encouraged to turn on their video, and recordings of the FGDs are taken in video format to enable recognition of non-verbal expression of cues. FGDs were transcribed verbatim by JL, with the names of the participants pseudo-anonymised to maintain confidentiality. Transcripts were not returned to participants for comment or correction, and no participant checking was conducted. We followed the COREQ guidelines for reporting qualitative research.^49^

### Data analysis

Quantitative data were tabulated and analysed using the Statistical Package for the Social Sciences (SPSS) version 28. Descriptive statistics were performed with a *p*-value of 0.05 as statistically significant. For the qualitative data, JL and BV coded the transcripts according to Braun and Clarke’s reflexive thematic analysis using NVivo 14 (QSR International Pty Ltd. Version 14, 2023). The agreement of theoretical sufficiency was discussed among JL and BV, which is the point where focus groups do not contribute new data to the understanding of the phenomenon.^50^ In our study, this saturation point was reached at the third focus group interview, where no new themes or insights were emerging from the discussions. Regular virtual meetings were held to collaboratively resolve differences in coding and reflect on the potential influences of our individual identities as students and educators during the active co-construction of our data and results,^51^ which enhances the rigour of our data interpretation.

### Reflexivity

JL, a male academic foundation doctor with experience in facilitating and participating in online CBL, brought a multifaceted perspective to the research as both a learner, clinician and educator. BV, a female PhD-trained scientist with expertise in qualitative research, conducted all the focus groups, ensuring consistency and rigour in data collection. No prior relationship was established between the participants and the researchers before the commencement of the study. Participants were informed only of the researcher’s role as a PhD-trained scientist conducting the focus groups for the purpose of academic research. Our diverse backgrounds allowed us to approach the data from different angles, helping to minimise individual biases. We actively sought to understand the influence of our professional roles on our interpretations through continuous critical reflection and dialogue.

## Results

### Participant demographics

Out of the 354 clinical year students, 160 completed the questionnaire (45% response rate). Students’ years of study influenced their perception of CR skills fostered during online CBL. Final-year students have significantly higher CR mean skills scores of 3.26 compared to third- and fourth-year students (*p* < 0.001). The preponderance of female participants (61.9%) reflects the demographics of local undergraduate medical students (see Table 1). In terms of focus groups, a total of 26 clinical-year medical students participated in three focus groups, including eight third-year, nine fourth-year, and eight final-year students. The focus groups ranged from eight to nine participants in size and lasted between 82 and 97 min (mean 93 min; total 4 h and 38 min).

**Table 1:** General information of the study participants (n = 160).

Variables	N (%)	Mean CR skills score (SD)	*p*-value
Years of study			
Year 3	88 (55.0%)	2.80 ± 0.06	<0.001^a^
Year 4	38 (23.8%)	2.98 ± 0.09	
Year 5	34 (21.2%)	3.26.± 0.11	
Gender			
Male	61 (38.1%)	2.98 ± 0.43	0.570^b^
Female	99 (61.9%)	2.92 ± 0.37	

a Kruskal-Wallis.

b Mann-Whitney.

Overall, students perceived online CBL to have fostered their CR ability (mean CR score = 2.94 ± 0.40), across the three domains: history taking, case discussions, and investigation and management, and significant differences are found between the domains (p=0.016), with CR skills related to case discussion found to be the most fostered through online CBL (mean score = 3.02 ± 0.48). Thirteen subthemes were constructed and grouped under three main themes: (i) mechanism of CR skills fostering during online CBL, (ii) barrier to CR fostering during online CBL, and (iii) recommendations to improve CR skills during online CBL. The results of the following section would be structured according to the CR domain, and quantitative results were presented in terms of the percentage agreements to each CR skills statement, while qualitative results were presented in terms of subthemes and selected quotes.

### History-taking domain

The majority of participants agreed that online CBL effectively fostered their history-taking skills, particularly in targeting questions to capture symptoms (93.7%) and taking the lead in history taking (86.3%). However, the ability to summarise patient information was the least developed skill, with only 58.8% feeling that it was adequately fostered. (see Table 2).

**Table 2:** Quantitative results on ‘Does online CBL help students foster their history-taking skills?’

Statements		Likert score^a^				
		Strongly disagree	Disagree	Agree	Strongly agree	Domain mean ± SD
A. History-taking domain						
1	I take the lead in the history taking in order to get the required information	1.87%	11.88%	58.75%	27.50%	2.98 ± 0.45
2	I target questions to capture symptoms which is considered important to specific causes of the symptoms.	0.62%	5.62%	48.13%	45.63%	
3	I ask questions in a logical order and not according to a checklist of questions.	1.25%	19.37%	54.38%	25.00%	
4	I am able to collect sufficient, high-quality data at reasonable speed.	1.88%	36.88%	54.37%	6.88%	
5	I summarise to check with the patient that my clinical thinking is based on correct information.	4.38%	36.88%	41.88%	16.88%	

a The responses to each statement were scored using a Likert scale ranging from 1 to 4 (strongly disagree, disagree, agree, and strongly agree).

In the focus group, participants cited that the virtual simulated environment encouraged them to ask more thoughtful questions and explore questions better from patients with complicated histories and praised that it is practical in real-life settings. The immediate feedback provided by lecturers during online CBL was highlighted as a significant advantage. This feedback helped students refine their questioning techniques and improve their history-taking skills (see Table 3, quotes 1–4). However, participants also reported significant challenges. They felt that online learning hindered their ability to perform physical examinations and develop effective communication skills (see Table 3, quotes 5 & 6). To enhance the effectiveness of online CBL in developing history-taking skills, participants recommended increasing the complexity of patient cases and incorporating actual patients into the simulations (see Table 3, quotes 7 & 8).

**Table 3:** Qualitative results for ‘How are history-taking skills fostered through online CBL?’

*Theme one: mechanism of CR skills fostering*	
*Practical simulated environment*	
1	*‘The online case-based discussions are better for me as it more of asking thoughtful structured questions while in real life, I tend to sway off to ask questions randomly which is not relevant or less important.’ – Year 4 student*
2	*‘We had two online simulated ward rounds, and some were role player patients with a complicated history, so I was able to explore questions better; it was very useful for my clinical reasoning skills.’ – Year 4 student*
3	*‘The virtual primary care consultation is very practical to real-life setting and is the closest to online learning of clinical reasoning skills.’ – Year 5 student*
*Immediate feedback*	
4	*‘One aspect that are really good about online case-based learning is the immediately feedback aspect. On Zoom, lecturer hear you phrase questions and immediately give you feedback, as opposed to when you are on the wards, you take history on your own and so does not learn the phrasing. I think the phrasing skill was a very good one that online CBL has facilitated when I do my mental health rotation completely online.’ – Year 3 student*
*Theme two: barrier to CR skills fostering*	
*Difficulties in physical examination*	
5	*‘When you learn through online, you tend to miss the aspects of technics on identifying signs during physical examination.’ – Year 4 student*
*Difficulties in communication skills training*	
6	*‘Communication skills is better by face-to-face learning as communicating with real patients are better.’ - Year 5 student*
*Theme three: recommendations to improve CR skills during online CBL*	
*Increase complexities of patient cases*	
7	*‘When you deal with complex patients, you would be able to gather lot more information when you explore on different angles and other hidden agenda.’ – Year 4 student*
*Utilising actual patients*	
8	*‘I think bringing real patients to Zoom is a step up than lecturers doing role play. I remember one lecturer did bring in actual patient for our end-of-placement assessment, I think it bring it closer to ward teaching, and it also trains you to stop asking questions that are based on an algorithm.’ – Year 3 student*

### Case discussion domain

The majority of participants agreed that online CBL effectively fostered their ability to list differential diagnoses (86.2%), identify the most probable diagnoses (83.8%), and identify risk factors (83.8%) during case discussions. However, the ability to identify clinical urgency was the least developed skill, with only 70.0% feeling that it was adequately fostered (see Table 4).

**Table 4:** Quantitative results on ‘Does online CBL help students foster their case-discussion skills?’

Statements		Likert score^a^				
		Strongly disagree	Disagree	Agree	Strongly agree	Domain Mean ± SD
B. Case discussion						
1	I am able to list out possible differential diagnoses.	0.00%	13.75%	63.75%	22.50%	3.02 ± 0.48
2	I am able to identify the most probable diagnosis with reasoning	0.00%	16.25%	58.75%	25.00%	
3	I am able to identify patient’s clinical urgency and stability	0.00%	30.00%	50.63%	19.37%	
4	I am able to identify the risk factors and precipitants associated with the diagnosis.	0.62%	15.63%	67.50%	16.25%	

a The responses to each statement were scored using a Likert scale ranging from 1 to 4 (strongly disagree, disagree, agree, and strongly agree).

Participants highlighted that online CBL sessions provided a conducive environment for in-depth group discussions, which they found beneficial for building their CR foundation before transitioning to real-life clinical settings. The relaxed online environment allowed for more focused and structured discussions without the pressures of face-to-face interactions (see Table 5, quotes 1 & 2). Despite these benefits, participants also faced significant challenges in online CBL. Engagement and focus were difficult to maintain, especially for those not directly involved in the discussion. Additionally, the variability in facilitator approaches, particularly those who favoured didactic over interactive sessions, hindered the development of CR skills (see Table 5, quotes 3–5). To address these challenges, participants recommended the use of online breakout rooms. These smaller, more interactive settings were seen as a way to enhance engagement and encourage student-led discussions, thereby improving CR skills (see Table 5, quotes 6 & 7).

**Table 5:** Qualitative results for ‘How are case discussion skills fostered through online CBL?’

*Theme one: mechanism of CR skills fostering*	
*In-depth virtual group discussion*	
1	*‘The online CBL classes allow us to discuss in-depth as the online environment is more conducive for discussion, more relax and is a good way to build up your foundation since it will remove those pressure you encounter during the face-to-face. It would be more beneficial to have your structure and foundation online and then you move on to clinical instead throwing yourself straight to clinical.’ – Year 4 student*
2	*‘During online CBL we don't practise examination skills, but primarily focus on practising CR, so we had more time to discuss, which is something I quite like in online teaching as opposed to in person.’ – Year 3 student*
*Theme two: barrier to CR skills fostering*	
*Difficulties in engagement and focus*	
3	*‘I think online learning depends on motivations during discussion. Not everyone in the group is keen to giving opinions or thoughts.’ – Year 3 student*
4	*‘It is hard for me to keep myself focused and involved throughout the whole session, especially when I am not the person taking history or participating.’ – Year 5 student*
*Facilitators variability*	
5	*‘It depends on the way the session is led by the facilitators. Sometimes it is a fully didactic session, only the facilitators would be talking, and there is a little group discussion to go about.’ – Year 4 student*
*Theme three: recommendations to improve CR skills during online CBL*	
*Utilise online breakout rooms*	
6	*‘‘Bad Day On Call’ is done on Microsoft Teams, using different breakout rooms simultaneously. Every student can participate in clerking patients, breaking bad news, managing patients, and submitting prescription forms. It is really helpful because we are always on our toes to manage the cases.’ – Year 5 student*
7	*‘The time allocated for student discussion in smaller breakout rooms is very useful as less time is wasted when student was asked by the lecturer to present as we had already discussed and formulated the answers beforehand.’ – Year 5 student*

### Investigation and management domain

The majority of participants agreed that online CBL effectively fostered their ability to establish management plans (83.8%) and determine necessary investigations (82.5%). However, the management skills least developed during online CBL were the ability to determine follow-up plans (54.4%) and assess progression and prognosis (55.6%) (see Table 6).

**Table 6:** Quantitative results on ‘Does online CBL help students foster their investigations and management skills?’

Statements		Likert score^a^				
		Strongly disagree	Disagree	Agree	Strongly agree	Domain Mean ± SD
C. Investigations and management						
1	I am able to identify and determine investigations.	1.25%	11.25%	67.50%	15.00%	2.87 ± 0.46
2	I am able to identify complications associated with the diagnosis, investigations and treatment.	1.87%	28.13%	60.63%	9.37%	
3	I am able to identify physical and psychosocial impact of the diagnosis.	0.63%	21.25%	56.25%	21.88%	
4	I am able to identify progression and prognosis associated with the diagnosis.	10.00%	40.63%	48.13%	7.50%	
5	I am able to establish management plans by taking into account of clinical guidelines.	1.25%	22.50%	61.25%	22.50%	
6	I am able to identify and determine a follow-up plan.	5.00%	40.62%	45.00%	9.38%	
7	I am able to identify my knowledge gaps and establish personal learning plans.	2.50%	35.62%	42.50%	19.38%	

a The responses to each statement were scored using a Likert scale ranging from 1 to 4 (strongly disagree, disagree, agree, and strongly agree).

Participants highlighted that online CBL provided valuable opportunities to practise conveying management plans to patients and to share knowledge sources with peers. These aspects of online CBL were particularly beneficial for developing CR skills in the domains of investigation and management, areas that can be challenging to address in face-to-face clinical teaching environments (see Table 7, quote 1 & 2). However, some participants found that online CBL cases often lacked realism and were overly simplistic. This limitation hindered their ability to develop comprehensive CR skills (see Table 7, quote 3). To address these issues and enhance CR skills, participants suggested the use of recorded consultations. These recordings could model interactions between senior clinicians and real-life patients, providing students with insights into history taking, patient management and communication skills (see Table 7, quote 4). Lastly, participants recommend quizzing metacognition to prompt CR process.

**Table 7:** Qualitative results for ‘How are investigation and management skills fostered through online CBL?’

*Theme one: mechanism of CR skills fostering*	
*Virtual patient explanation*	
1	*‘Online case-based discussions help me develop my clinical skills in the management aspect, for example, explaining the management to patient and reason it out, which you don’t get in the busier hospital settings.’ – Year 5 student*
*Virtual information sharing*	
2	*‘As for the online CBL, the technology aspect has actually helped us a lot. We are able to share information through our screen, play or send videos or any link which are relevant, in comparison we can't have the face-to-face teaching as there are not many locations to do so.’ – Year 4 student*
*Theme two: barrier to CR skills fostering*	
*Unrealistic patient cases*	
3	*‘Online CBL is unrealistic because they usually come with one problem only, and that's all we have to deal with, whereas in real life that is not the case, where the patients have a whole list of problems and complications.’ – Year 5 student*
*Theme three: recommendations to improve CR skills during online CBL*	
*Utilise recordings of patient–clinician interactions*	
4	*‘Recordings of primary care consultation is helpful for building clinical reasoning skills as we could see how general practitioners would take the history, manage patients’ expectations, communication skills and handling patients in their own individual way. We would watched the video with lecturers. The lecturers would pause the videos at certain points and ask us to think what would be the first thought that comes to our mind when the patients mention their symptoms. Perhaps the university could look into the possibility of incorporating this platform in our curriculum.’ – Year 4 student*
*Active quizzing*	
5	*‘I think it is a very important job for the online facilitator to probe you to ask why do you think this is the differential, why do you do this investigation. Is there anything else you would like to do. I found that when my lecturer does that, it probes my clinical reasoning and makes me to think more.’ – Year 5 student*

## Discussion

### Summary of key findings

This is the first study that provides a comprehensive evaluation of CR fostering during online CBL in clinical-year medical students. Online CBL is perceived to have fostered students’ CR skills, with final-year students reporting a higher CR ability during online CBL compared to more junior medical students. We also found significant differences between CR domains, with the case-discussion domain being perceived as the most fostered through online CBL, followed by the history-taking and management domain. Thematic analysis of the focus groups identified the mechanisms of, barriers to and recommendations for CR skills fostering during online CBL.

### Comparison with existing literature

Our study builds on previous research suggesting the benefits of online CBL^8^^,^^41^ and adds a novel perspective by elucidating the explicit mechanisms through which CR skills are fostered. Traditional in-person teaching, such as the presentation of case histories and discussions on ward rounds and in clinics, has always been considered the gold standard as it enables students to engage with real patients, which fosters practical, hands-on learning and real-world variations in patient presentations that are essential for nuanced decision-making.^7^ However, traditional clinical settings are often constrained by patient availability, time pressures, and the unpredictable nature of ward-based learning.^52^ These factors can limit the consistency and equity of learning opportunities for large student cohorts, with some students receiving less exposure to critical cases or less time for detailed case discussions and feedback. In contrast, online CBL overcomes these limitations by offering a scalable and standardised approach, making it a valuable complement to traditional methods, especially in educational contexts involving large cohorts.^53^

Participants reported that their CR skills improved through better exploration of questions during history taking with simulated patients and receiving immediate feedback on their questioning techniques from lecturers. This finding aligns with literature indicating that virtual clerkships enhance CR skills development through direct patient interaction and feedback from tutors and peers.^13^ Additionally, participants mentioned that the simulated online environment provided a sense of security, enhancing their self-efficacy in conversing with simulated patients and improving their CR skills.^5^^,^^6^ Participants noted that online CBL encourages in-depth and focused case discussions, providing opportunities for patient information-giving and peer information-sharing. These advantages are attributed to the online environment, which fosters CR skills due to a structured, secure environment for detailed case discussions and feedback. This contrasts with the fast-paced real-life clinical settings, which may hinder CR skills cultivation.^54^ Furthermore, online CBL allows medical students to learn about rare medical conditions that are less commonly encountered in clinical settings. This approach helps overcome the limitations of in-person clinical training, such as the rarity of certain diseases, local patient demographics, and resource availability, thereby broadening their CR skills.^52^

However, participants also encountered barriers to CR skills development during online CBL, such as unrealistic, textbook-like case presentations. The complexity and unique presentations of patients are necessary to challenge students and help them develop their history-taking and management-related CR skills.^54^ Engagement and focus issues were also noted as barriers, with interactivity being crucial for CR skills development. This aligns with the findings of Geha *et al*^14^ that interactive sessions between teachers, students and peers are key to student engagement. Mukhtar *et al*^6^ also emphasised the importance of teacher–student interactivity in fostering CR skills.

To improve interactivity, educators should encourage collaborative CBL with discussions in small breakout rooms. Student-led discussions were found to promote participation,^55^ collaborative thinking, active idea sharing, CR efficiency and assessment scores.^34^^,^^35^ Pre-assigning discussion topics and using breakout rooms for smaller group collaboration were also recommended to improve engagement and participation, and leveraging the positive effects of collaborative learning to enhance CR skills.^39^ Educators are also encouraged to facilitate group viewing of virtual recorded actual patient encounters with expert commentary, which literature supports as enhancing interactivity and educational value.^56^ Interactive web-based cases supplemented by video consultations were also recommended to increase student engagement and exposure to a variety of conditions.^19^ Literature suggests that patient video cases are successfully used in many specialties, increasing student exposure to a wider variety of patients and providing CR skills practice opportunities.^20^ Our data correlated with those of Sutherland *et al*,^40^ where the probing and coaching of students’ diagnostic reasoning during online CBL are recommended as an effective teaching strategy. This could encourage self-explanation, where learners make connections and elaborate by explaining their thinking and rationale.^57^ Lastly, educators making their own CR process visible and explaining their thought processes were also found to be particularly helpful^35^ in facilitating students’ CR skills during online CBL.

### Methodological strengths and limitations

One strength of this study is the comprehensive evaluation of CR fostering during online CBL using a mixed-methods approach, which allows for a more nuanced understanding of the perceived benefits and challenges of online CBL. Additionally, the use of focus groups provided deeper insights into the mechanisms through which CR skills are developed and the barriers faced by students. There are limitations that are worth considering while interpreting our findings. One limitation of this study is that the single university involvement in a primarily Asian context may have limited the generalisability of the study to a Western context. Secondly, a response rate of 46% may have limited the power of the study and resulted in potential response bias. Lastly, while self-reported data provide valuable insights into student perceptions, it may introduce bias and may not accurately reflect their ability to apply these skills in real-world clinical settings. Therefore, future research should prioritise validating these perceived improvements through objective measures, such as structured assessments like objective structured clinical examinations (OSCEs). Despite these limitations, our findings fill a gap in the literature by offering a better understanding of the role of online CBL in CR fostering from students’ perspectives. The study population captures junior to senior students, implying that online CBL is effective for students of all stages of clinical experience and career.

### Implications for future research

As Donkin, Yule and Fyfe^8^ noted, the use of online CBL in health professionals’ education is expanding rapidly alongside the adoption of new technologies. To ensure the adoption of best evidence-based practices internationally, we encourage researchers to investigate the experiences of educators involved in delivering online CBL, which would provide valuable insights into its practical application and offer perspectives on the time, resources and capacity required for online CBL, especially in comparison to traditional teaching. Additionally, research should focus on how educators can be better supported to fully realise the potential of online CBL. Another critical area for future research is the exploration of blended learning approaches that integrate both online and face-to-face modalities in CR fostering. Gaining a clearer understanding of educators’ perspectives on the scalability, resource requirements and optimal balance of digital and in-person education will be key to guiding the successful integration of these methods into health profession curricula.

## Conclusion

This study provides valuable insights into how online CBL can support the development of CR skills among medical students. While students identified several benefits, such as improved question exploration and immediate feedback, challenges like unrealistic case presentations and engagement issues were also highlighted. Recommendations to enhance case complexity, incorporate real patient simulations, and increase interactivity through breakout rooms and recorded consultations offer potential ways to optimise online CBL. However, further research is essential to develop objective assessments of students’ CR skills and to evaluate the long-term impact of online CBL. This will be critical to inform its broader integration into health profession curricula. We encourage educators to adopt a blended learning approach that leverages the unique strengths of both traditional and online methods to provide a more comprehensive strategy for developing CR skills. Continuous exploration of innovative methods will also be essential to enhance CR skill acquisition further.

## Data availability

The datasets generated and/or analysed during the current study are not publicly available due to privacy and ethical concerns, but are available from the corresponding author on reasonable request.

## Funding statement

This research did not receive any specific grant from funding agencies in the public, commercial, or not-for-profit sectors.

## Ethics approval and consent to participate

Ethical approval was granted by the Research, Policy, Intelligence and Ethics Team, Newcastle University Research Office, case number Ref: 11486/2020. All participants provided written consent.

## CRediT authorship contribution statement

**Jun Jie Lim:** Writing – review & editing, Writing – original draft, Visualization, Validation, Software, Resources, Project administration, Methodology, Investigation, Formal analysis, Data curation. **Bhavani Veasuvalingam:** Writing – review & editing, Writing – original draft, Visualization, Validation, Supervision, Resources, Project administration, Methodology, Investigation, Formal analysis, Data curation, Conceptualization.

## Declaration of competing interest

The authors declare that they have no known competing financial interests or personal relationships that could have appeared to influence the work reported in this paper.

## Appendix Supplementary materials

Image, application 1

Image, application 2
