# Supplementary material for: Does online case-based learning foster clinical reasoning skills? A mixed-methods study
Source: Future Healthc J. 2024 Nov 13;12(1):100210. doi: 10.1016/j.fhj.2024.100210 (PMC11625323; doi:10.1016/j.fhj.2024.100210)

**Appendix A – Final Questionnaire**

**Part 1: History taking**

1. I take the lead in the history taking in order to get the required information.


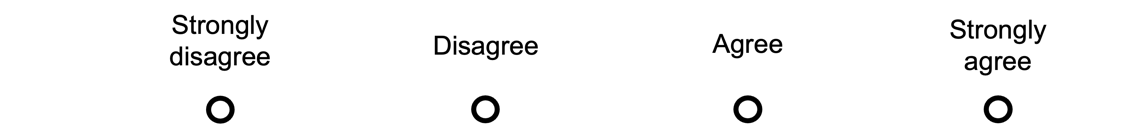


1. I target questions to capture symptoms which is considered important to specific causes of the symptoms.


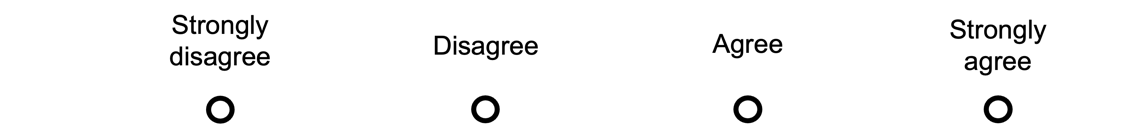


1. I ask questions in a logical order and not according to a checklist of questions.


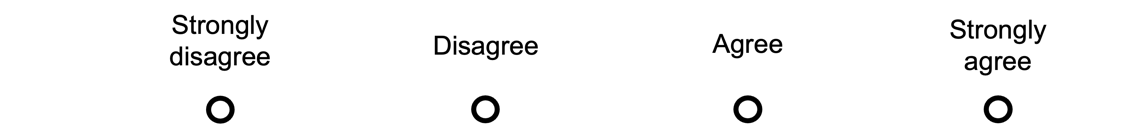


1. I am able to collect sufficient, high quality data at reasonable speed.


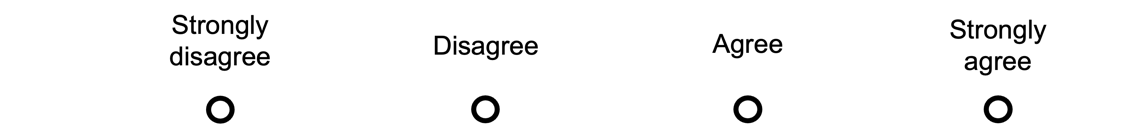


1. I summarise to check with the patient that my clinical thinking is based on correct information.


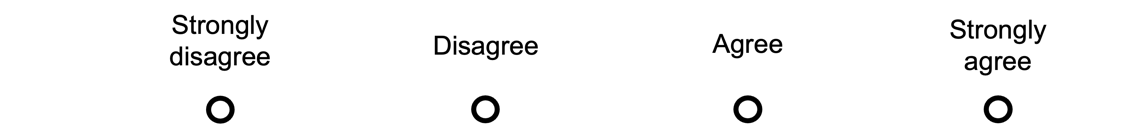


**Part 2: Case discussion**

1. I am able to list out possible differential diagnosis.


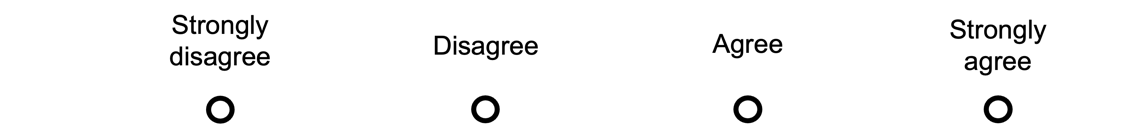


1. I am able to identify the most probable diagnoses with reasoning.


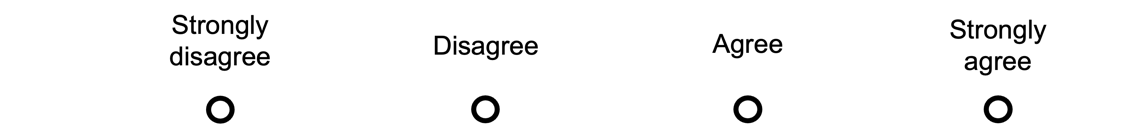


1. I am able to identify patient's clinical urgency and stability.


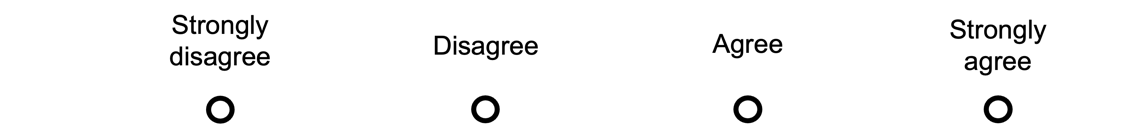


1. I am able to identify the risk factors and precipitants associated with the diagnosis.


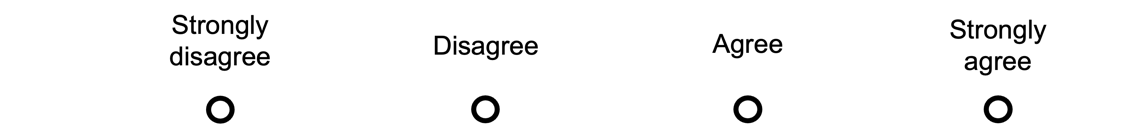


**Part 3: Investigations and management**

1. I am able to identify and determine investigations.


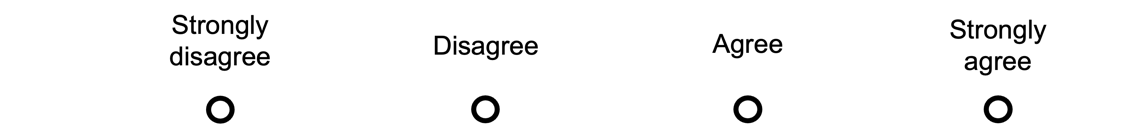


1. I am able to identify complications associated with the diagnosis, investigations and treatment.


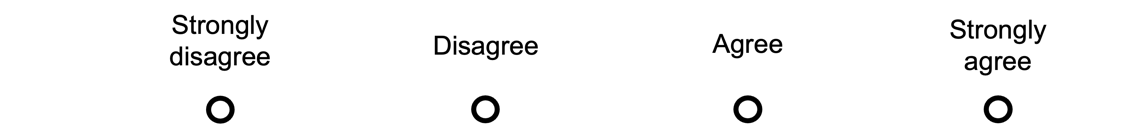


1. I am able to identify physical and psychosocial impact of the diagnosis.


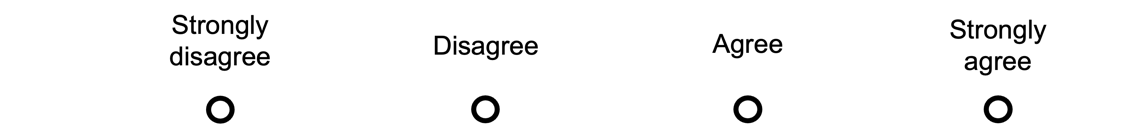


1. I am able to identify progression and prognosis associated with the diagnosis.


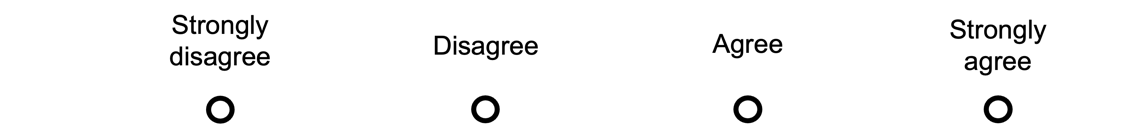


1. I am able to establish management plans by taking into account of clinical guidelines.


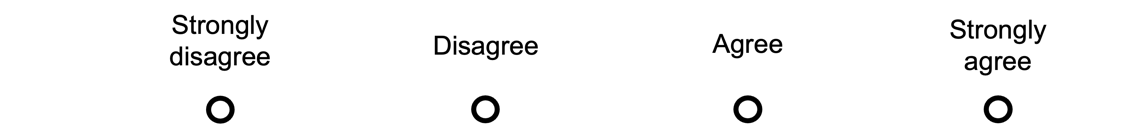


1. I am able to identify and determine a follow-up plan.


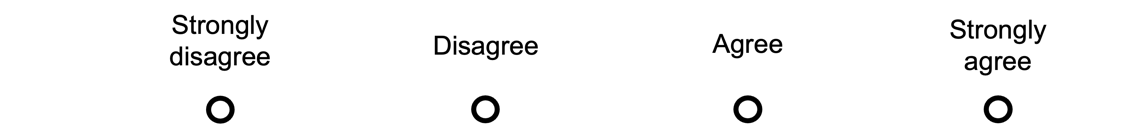


1. I am able to identify my knowledge gaps and establish personal learning plans.


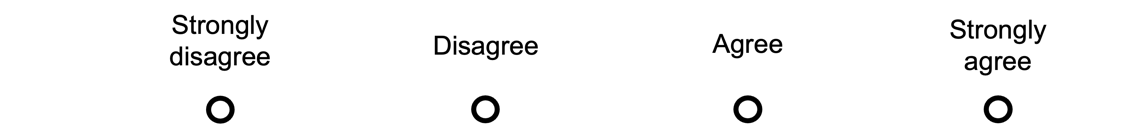

Supplement: Supplementary file 1 [file mmc1.docx]
