# Supplementary material for: Does online case-based learning foster clinical reasoning skills? A mixed-methods study
Source: Future Healthc J. 2024 Nov 13;12(1):100210. doi: 10.1016/j.fhj.2024.100210 (PMC11625323; doi:10.1016/j.fhj.2024.100210)
Supplement: Supplementary file 2 [file mmc2.docx]

**Appendix B - Final Semi-Structured Focus Group Interview Guide**

1. Can you describe your overall experience with online case-based learning in the context of developing your clinical reasoning skills?
2. How do you think online case-based learning compares to traditional in-person learning in terms of its effectiveness in fostering your clinical reasoning skills?
3. What specific aspects of the online environment do you find most beneficial for developing your clinical reasoning skills? Can you provide examples?
4. Can you describe any challenges you have faced when trying to perform clinical reasoning through online case-based learning?
5. Based on your experience, what changes or improvements would you recommend to enhance the effectiveness of online case-based learning for developing clinical reasoning skills?
